# Multidrug-resistant tuberculosis (MDR-TB) strain infection in macaques results in high bacilli burdens in airways, driving broad innate/adaptive immune responses

**DOI:** 10.1038/s41426-018-0213-z

**Published:** 2018-12-12

**Authors:** Enzhuo Yang, Rui Yang, Ming Guo, Dan Huang, Wandang Wang, Zhuoran Zhang, Crystal Chen, Feifei Wang, Wenzhe Ho, Ling Shen, Heping Xiao, Zheng W. Chen, Hongbo Shen

**Affiliations:** 1 Clinic and Research Center of Tuberculosis, Shanghai Key Laboratory of Tuberculosis, Shanghai Pulmonary Hospital, Tongji University School of Medicine, Shanghai, China; 2 Department of Microbiology & Immunology and Center for Primate Biomedical Research, University of Illinois College of Medicine, Chicago, IL USA; 3 Unit of Anti-Tuberculosis Immunity, Institut Pasteur of Shanghai, Chinese Academy of Sciences, Shanghai, 200031 China; 4 College of Medicine,Wuhan University, Wuhan, Hubei Province 430072 China; 5 Department of Medical Microbiology and Parasitology, Shanghai Medical College, Fudan University, Shanghai, 200032 China

## Abstract

Tuberculosis (TB) has become the most deadly infectious diseases due to epidemics of HIV/AIDS and multidrug-resistant/extensively drug-resistant TB (MDR-/XDR-TB). Although person-to-person transmission contributes to MDR-TB, it remains unknown whether infection with MDR strains resembles infection with drug-sensitive (DS) TB strains, manipulating limited or broad immune responses. To address these questions, macaques were infected with MDR strain V791 and a drug-sensitive Erdman strain of TB. MDR bacilli burdens in the airway were significantly higher than those of the Erdman control after pulmonary exposure. This productive MDR strain infection upregulated the expression of caspase 3 in macrophages/monocytes and induced appreciable innate-like effector responses of CD3-negative lymphocytes and Ag-specific γδ T-cell subsets. Concurrently, MDR strain infection induced broad immune responses of T-cell subpopulations producing Th1, Th17, Th22, and CTL cytokines. Furthermore, MDR bacilli, like the Erdman strain, were capable of inducing typical TB disease characterized by weight loss, lymphocytopenia, and severe TB lesions. For the first time, our results suggest that MDR-TB infection acts like DS to induce high bacterial burdens in the airway (transmission advantage), innate/adaptive immune responses, and disease processes. Because nonhuman primates are biologically closer to humans than other species, our data may provide useful information for predicting the effects of primary MDR strain infection after person-to-person transmission. The findings also support the hypothesis that a vaccine or host-directed adjunctive modality that is effective for drug-sensitive TB is likely to also impact MDR-TB.

## Introduction

Tuberculosis (TB), which is caused by *Mycobacteria tuberculosis* (Mtb), has recently become the leading cause of infectious disease-related mortality worldwide due to multidrug-resistant TB (MDR-TB) epidemics and the HIV/AIDS pandemic^1^. MDR-TB is defined as a form of TB caused by bacteria that are resistant to at least the two most powerful first-line anti-TB drugs, isoniazid (INH) and rifampicin (RIF). Extensively drug-resistant TB (XDR-TB) is caused by bacteria that are resistant to INH, RIF, and the following main second-line anti-TB drugs: fluoroquinolones and aminoglycosides^2,3^. MDR-TB/XDR-TB can result from either inadequate use of anti-TB drugs in TB patients or person-to-person transmission of MDR/XDR strains. MDR-TB/XDR-TB are now occurring at high rates^4^. Success rates for treatment of MDR-TB/XDR-TB are low due to limited treatment options, delayed diagnosis, and inadequate health care infrastructure, and MDR-TB/XDR-TB infections result in a large proportion of global TB-related mortality^5,6^. The World Health Organization (WHO) and other funding agencies, including the National Institutes of Health (NIH), have declared MDR-TB/XDR-TB an emergency and called for better diagnosis, treatment, and prevention, as well as an increased effort to understand the pathogenesis of TB. Elucidating the pathogenesis of and immune responses to MDR-TB/XDR-TB is of considerable importance for designing effective adjunctive therapy, given the lack of effective anti-TB drugs.

In vitro studies have suggested that MDR-TB and drug-sensitive (DS) strains can exhibit different growth and replication kinetics due to the fitness costs of drug resistance^7–9^. However, little is known about whether such differences between MDR-TB and DS strains influence in vivo infections or their consequences in humans or nonhuman primates (NHPs). In fact, there is a lack of reports describing the progression of initial MDR Mtb infections to pathological consequences in humans and NHPs.

Mtb and humans have been coevolving for >50,000 years. While T-cell responses that develop at the right time and in the right location may confer protective immunity, Mtb can efficiently manipulate overreactive host responses to create infectious lesions in the lung for its survival and spread^10^. However, whether MDR strains act like DS strains, manipulating hosts to generate infectious TB lesions that facilitate transmission remains poorly documented.

It has recently been estimated that the person-to-person transmission is the major cause of the global epidemic of MDR-TB/XDR-TB^11^. However, it remains unclear whether a primary MDR-/XDR-TB infection resembles a DS TB infection. The absence of knowledge is largely attributed to the fact that most patients do not come to clinics until Mtb infection has progressed to full-blown TB disease. However, whether a healthy human scan mount limited or broad immune responses after their first exposure to MDR-TB/XDR-TB bacilli has not been reported. In this context, failures in immune responses to MDR-TB/XDR-TB have not been elucidated in humans. These questions cannot be adequately addressed without appropriate animal models^12^.

Nonhuman primates resemble humans in terms of their biology and immune responses, and cynomolgus and rhesus macaques have served as useful models for studies of human TB^12–17^. In the current study, we investigated MDR-TB and DS Erdman infections and host immune responses using cynomolgus macaques.

## Results

### MDR strain V791 (MDR-Mtb) disseminated to the airway more productively than Erdman strain in the early infection process

The hallmark of Mtb infection in humans is the creation of infectious lesions or niches in the lung that facilitate the survival and transmission of the bacilli. Currently, it remains unknown whether pulmonary exposure to MDR-Mtb bacilli in humans and NHPs could more rapidly initiate replication or productive infection than exposure to DS TB strain. Addressing this question can determine potential differences in replication between the in vivo setting and in vitro culture^18^ and will help to elucidate the pathogenesis of MDR-Mtb. Thus, two groups of cynomolgus macaques were similarly infected with either the MDR strain V791 or DS Erdman strain, through the bronchoscope-guided dissemination of 500 CFU to the right caudal lobe^19–21^. The MDR-TBV791 strain (MDR-Mtb), a clinical isolate from a MDR-TB patient, exhibited resistance to the clinical TB drugs INH, RIF, streptomycin (SM), and ofloxacin (OFX)^3^(Table 1).

**Table 1:** MDR-Mtb-V791, a clinical isolate from a MDR-TB patient (Clinical ID 20120824), exhibited resistance to clinical TB drugs

Drugs	Drug concentrations (μg/ml)	CFU (×10e7)			Drug-resistant percentage^a^
Isoniazide (INH)	0.2	>300	>300	414	≧1%
	1	306	263	295	≧1%
	10	0	0	–	≦1%
Rifampicin (RIF)	5	197	227	222	≧1%
	25	3	7	–	≧1%
	50	0	0	–	≦1%
Streptomycin (SM)	2	329	345	303	≧1%
	10	279	319	297	≧1%
	100	256	261	–	≧1%
Ofloxacin (OFX)	2	247	243	–	≧1%
	10	0	0	–	≦1%
Ethambutol (EMB)	2	234	221	–	≧1%
	5	97	109	–	≧1%
	50	0	0	–	≦1%
Control		196	226	258	

^a^Drug-resistant percentage = CFU of Mtb on 7H11 plates with drugs/CFU of Mtb on 7H11 plates without drugs

**Fig. 1:**
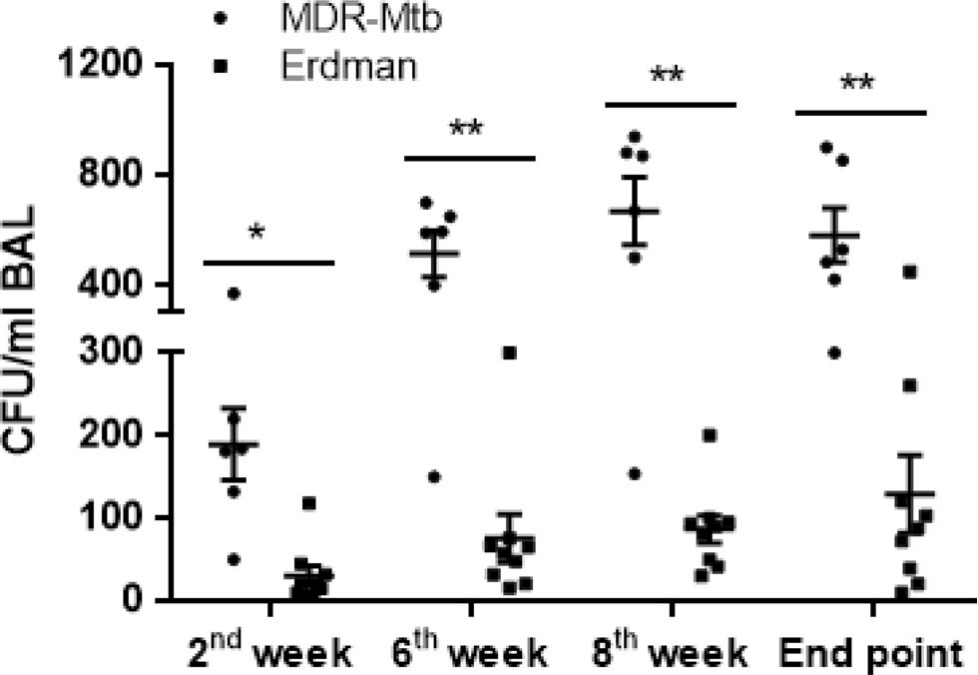
MDR-Mtb disseminated to the airway more effectively than Erdman-Mtb during the early infection process. Data are represented as the median number ± SEM of *M. tuberculosis* bacilli in the BALF at different time points after pulmonary infection that was introduced by bronchoscope-guided inoculation of MDR-Mtb and Erdman-Mtb. ^***^p < 0.001, ^**^p < 0.01, ^*^p < 0.05, according to nonparametric comparisons between groups infected with MDR-Mtb (*n* = 6) and Erdman-Mtb (*n* = 9)

TB bacilli burdens in the airway were measured by CFU counts in bronchoalveolar lavage fluid (BALF) collected after infection with the Erdman and MDR-Mtb strains. Interestingly, during the early infection process, the mean CFU counts (second week) in the BALF from MDR-Mtb-infected macaques were almost five times higher than those in the BALF from Erdman-infected animals (Fig. 1). Moreover, MDR-Mtb bacilli subsequently sustained their greater abundance in the airway at every time point compared with the Erdman strain (Fig. 1).

Thus, these results suggest that MDR-Mtb appeared to replicate and spread in the airway more readily than the Erdman strain in the early pulmonary infection process.

### Early productivity of the MDR-Mtb infection drove caspase 3 production in macrophages/monocytes and manipulated effector responses of innate-like lymphocytes, including γδT cells

**Fig. 2:**
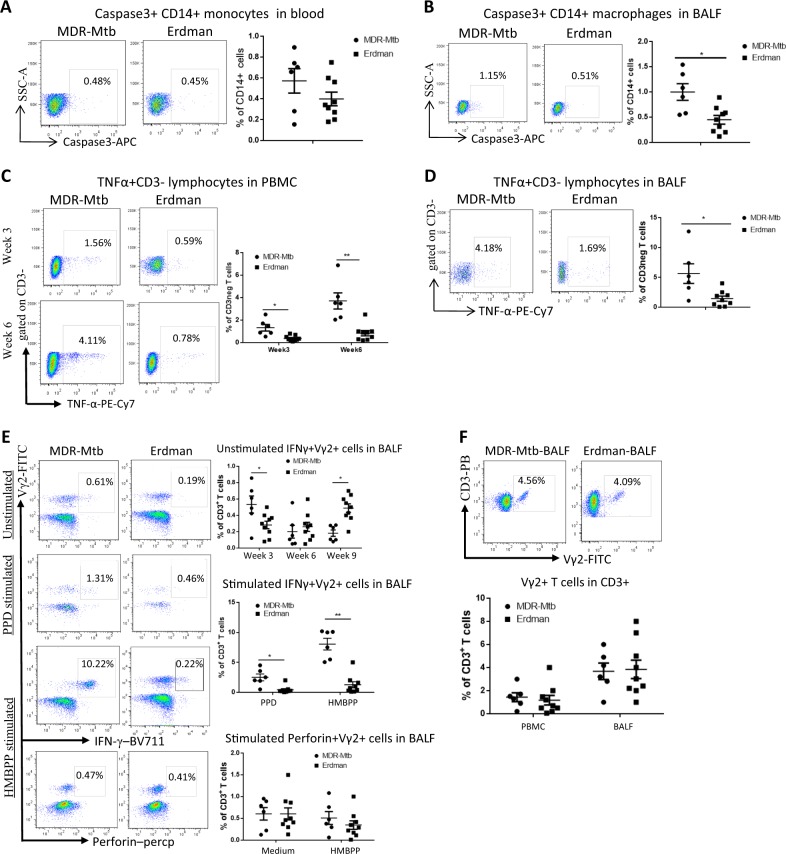
Early productivity of the MDR-Mtb infection drove caspase 3 production in monocytes/macrophages and induced effector responses of innate-like lymphocytes, including TB phospho antigen-specific γδ T cells. **a** Representative dot plots of caspase 3+ CD14+ monocyte profiles in the blood at week 3 from MDR-Mtb- and Erdman-Mtb-infected macaques. The population containing surrogate monocytes was gated in the standard size/light scatters. Baseline levels of caspase 3+ CD14+ monocytes/macrophages in the blood or BALF were <0.1% (data not shown). **b** Data are represented as the mean percentages of caspase 3+ CD14+ macrophages in the BALF from MDR-Mtb- and Erdman-Mtb-infected macaques in week 3. The population containing surrogate macrophages was gated in the standard forward/light scatters. **c** Representative flow plots show TNF-α+ CD3− effector lymphocytes in the blood from MDR-Mtb- and Erdman-Mtb-infected macaques in weeks 3 and 6. **d** Representative flow plots show TNF-α+ CD3− lymphocytes in week 6 in the BALF from MDR-Mtb- and Erdman-Mtb-infected macaques. **e** Representative flow plots show CD3+ Vγ2+ T effector cells in the BALF from MDR-Mtb- and Erdman-Mtb-infected macaques. The upper panels show Vγ2+ T cells constitutively producing IFN-γ in the absence of the antigen stimulation of cells in the BALF collected in weeks 3, 6, and 9. The baseline levels of constitutive IFN-γ+ Vγ2+ T cells before infection were <0.01–0.1%. The middle panels show the mean frequencies of CD3+ Vγ2+ IFN-γ+ T effector cells after PPD or HMBPP stimulation of BALF cells collected in week 3. The values measured in the medium alone were subtracted from the experimental measurements. The lower panel shows the mean percentage of CD3+ Vγ2+ perforin+ T cells after medium or HMBPP stimulation of BALF cells collected in week 3. Vγ2+ T effector cells were measured by ICS as previously described. Data were derived from up to six MDR-Mtb-infected macaques and nine Erdman-Mtb-infected macaques. ^**^p < 0.01, ^*^p < 0.05. **f** Representative flow histograms show the frequencies of CD3+ Vγ2+ T cells in PBMCs and the BALF in week 3. Data are represented as the mean + ± SEM of the percentages (%) and were analyzed by the Mann–Whitney test (nonparametric method)

We then sought to examine whether the high MDR-Mtb burdens detected in the early phase would lead to apparent innate immune responses. To this end, we first measured caspase 3 production by macrophages/monocytes as a marker of apoptotic or necrotic processes or innate immune responses, as caspase 3 is a downstream execution protease involved in both apoptosis and necrosis^22^. We found that although the increased production of caspase 3 by blood monocytes did not reach statistical significance (Fig. 2a), by week 3, the number of caspase 3+ CD14+ macrophages in the BALF of MDR-Mtb-infected macaques was significantly greater than the number in the BALF from Erdman strain-infected animals (Fig. 2b). At the same time, we also measured TNF-α production, as a TNF-α response co-occurring with caspase 3 production is not only the result of TB-driven immune activation, but also the facilitator of apoptosis and necrosis^23,24^. We found that compared with infection with the Erdman strain, MDR-Mtb infection induced the production of more TNF-α by innate-like CD3 lymphocytes in the blood at weeks 3 and 6 and in the BALF at week 6 (Fig. 2c, d); however, the same increased production was not observed for CD3+ T cells, including γδ T cells (data not shown) at weeks 3 and 6.

Because γδ T cells, like certain CD3 lymphocytes, may also act as innate-like lymphocytes, responding faster than adaptive immune cells, we investigated Mtb phospho antigen-specific Vγ2Vδ2 T-cell subsets in macaques infected with MDR-Mtb and Erdman strain. We detected more pronounced effector responses of IFN-γ-producing Vγ2+ T cells during primary MDR-Mtb infection than during infection with the Erdman strains (Fig. 2e), although the percentages of Vγ2+ T cells at week 3 were comparable between the two groups of macaques (Fig. 2f). Notably, the ability of Vγ2+ T cells in the airway to produce IFN-γ at week 3 was greater after MDR-Mtb infection than after infection with the Erdman strain with regard to each of the following: (i) “constitutive” production of IFN-γ without in vitro antigen stimulation (Fig. 2e upper panel); (ii) production of IFN-γ after in vitro PPD stimulation (Fig. 2e middle panel, Supplementary Figure); and (iii) production of IFN-γ after phospho antigen HMBPP stimulation (Fig. 2e middle panel). However, we found no significant difference in the production of perforin by Vγ2+ T cells at week 3 between the two groups of macaques (Fig. 2e lower panel).

Taken together, these results suggested that compared with infection with the Erdman strain, early productivity of the MDR-Mtb infection resulted in increased caspase 3 production by macrophages/monocytes and more apparent effector responses of innate-like γδ T cells and CD3-negative lymphocytes.

### Early high MDR-Mtb burdens and innate-like lymphocyte effectors coincided with the development of broad adaptive T-cell responses

**Fig. 3:**
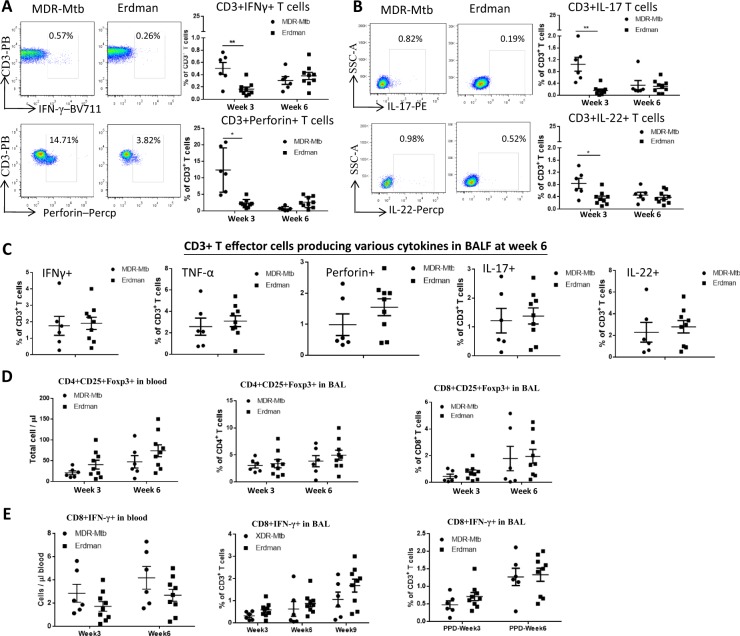
Early high MDR-Mtb burdens and innate-like lymphocyte effectors coincided with the development of broad adaptive T-cell responses. **a** Representative flow cytometry data (left) show percentages of CD3+ T effector cells producing IFN-γ (upper panel) and perforin in PBMCs from MDR-Mtb- and Erdman-infected macaques at weeks 3 and 6, respectively. Data are represented as the mean ± SEM. **b** Representative flow cytometry data (left) show percentages of CD3+ T effector cells producing IL-17 (upper panel) or IL-22 in PBMCs at weeks 3 and 6, respectively. Data are represented as the mean ± SEM. **c** The data represent the mean percentages ± SEM of CD3+ T effector cells producing IFN-γ and perforin, TNF-α, IL-17, and IL-22 in BALF at week 6. **d** The data show the mean numbers or percentages ± SEM of CD4+ CD25+ Foxp3+ T cells in blood and BAL and those of CD8+ CD25+ Foxp3+ T cells in BAL at weeks 3 and 6 after infection. **e** Data are represented as the mean numbers or percentages ± SEM of CD8+ IFN-γ+ T cells in blood and BALF measured by ICS with or without PPD stimulation at different time points. Similar data trends for CD4+ IFN-γ+ T cells in blood and BALF measured by ICS with or without PPD stimulation (data not shown). Data were derived from up to six MDR-Mtb-infected and nine Erdman-infected macaques and were analyzed by the Mann–Whitney test (nonparametric method). ^*^p < 0.05, ^**^p < 0.01

Whether MDR-Mtb infection induces limited or broad responses by T-cell subpopulations has not been investigated. We therefore sought to examine whether the early productivity of the MDR-Mtb infection and the innate immune responses could also result in the development of adaptive immune responses by T cells. We took advantage of the useful direct ICS assay without Ag stimulation in vitro to measure the effector responses of the αβ T-cell subpopulations. The direct ICS approach has recently been validated for the detection of large numbers of T effector cells constitutively producing broad-spectrum cytokines in macaques infected with TB or other pathogens^13,17,25–27^ and in patients with TB^28^ in comparison with controls^21,29,30^. Using the direct ICS approach, we found that macaques infected with MDR-Mtb exhibited more substantial increases in T effector cells constitutively producing IFN-γ, perforin, IL-17, and IL-22 in the blood at week 3 after infection when compared with the Erdman-infected animals (Fig. 3a, b). However, such enhanced and broad T effector responses appeared to be transient because similar levels of T effector cells were detected at week 6 between MDR-Mtb- and Erdman-infected macaques (Fig. 3a, b). Moreover, the pulmonary responses of the effector cells producing the Th1, Th17/Th22, and CTL cytokines were comparable between the two groups (Fig. 3c). In contrast to the early difference in T effector responses, however, CD3+ CD4+ CD25+ Foxp3+ cells, and CD8+ IFN-γ+ cells were comparable at all time points between these two groups of macaques (Fig. 3d, e).

Thus, these results suggested that MDR-Mtb infection of macaques could induce broad adaptive responses of T effector cells constitutively producing IFN-γ, perforin, IL-17, and IL-22.

### MDR-Mtb and Mtb Erdman shared the ability to induce clinical TB disease and typical TB pathology in NHPs

It remains unclear whether a primary MDR-TB infection after person-to-person transmission of bacilli resembles infection by DS Mtb^11^. To address this question, the clinical progression of TB infection was assessed in MDR-Mtb- and Erdman-infected macaques^3,19–21^.

**Fig. 4:**
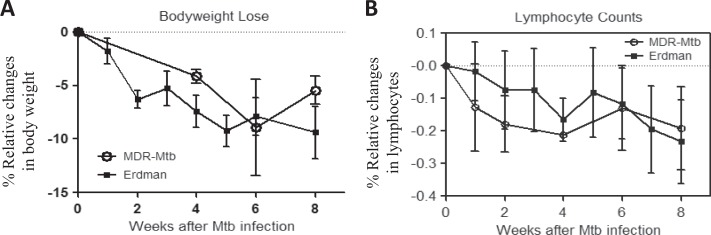
MDR-Mtb and Erdman-Mtb shared the ability to induce clinical TB manifestations in nonhuman primates. **a** Percent changes in body weights after infection compared to the baseline values. **b** Percent changes in blood lymphocyte counts after infection compared to the baseline values. Note that there were no significant differences in body weight or lymphocyte count between MDR-Mtb-infected and Erdman-Mtb-infected macaques. The data are represented as the mean ± SEM and were analyzed by the Mann–Whitney test (nonparametric method)

We focused on the clinical aspects of weight loss and a decreased number of circulating lymphocytes (lymphocytopenia), as we and others have demonstrated that these two parameters represent prominent clinical manifestations in Mtb-infected macaques^20,21^. We found that similar to infection with the Erdman strain, the infection of macaques with MDR-Mtb induced clear weight loss (Fig. 4a) and lymphocytopenia (Fig. 4b). Furthermore, no significant differences in the extent of the weight lost or the degree of lymphocytopenia were observed between macaques infected with MDR-Mtb and those infected with the Erdman strain (Fig. 4a, b).

Finally, we sought to examine whether MDR-Mtb could induce infectious TB lesions or pathology similar to those induced by the Erdman strain after high-dose 500 CFU infection of NHPs. We conducted complete necropsies and bacterial pathology studies, as previously described.

**Fig. 5:**
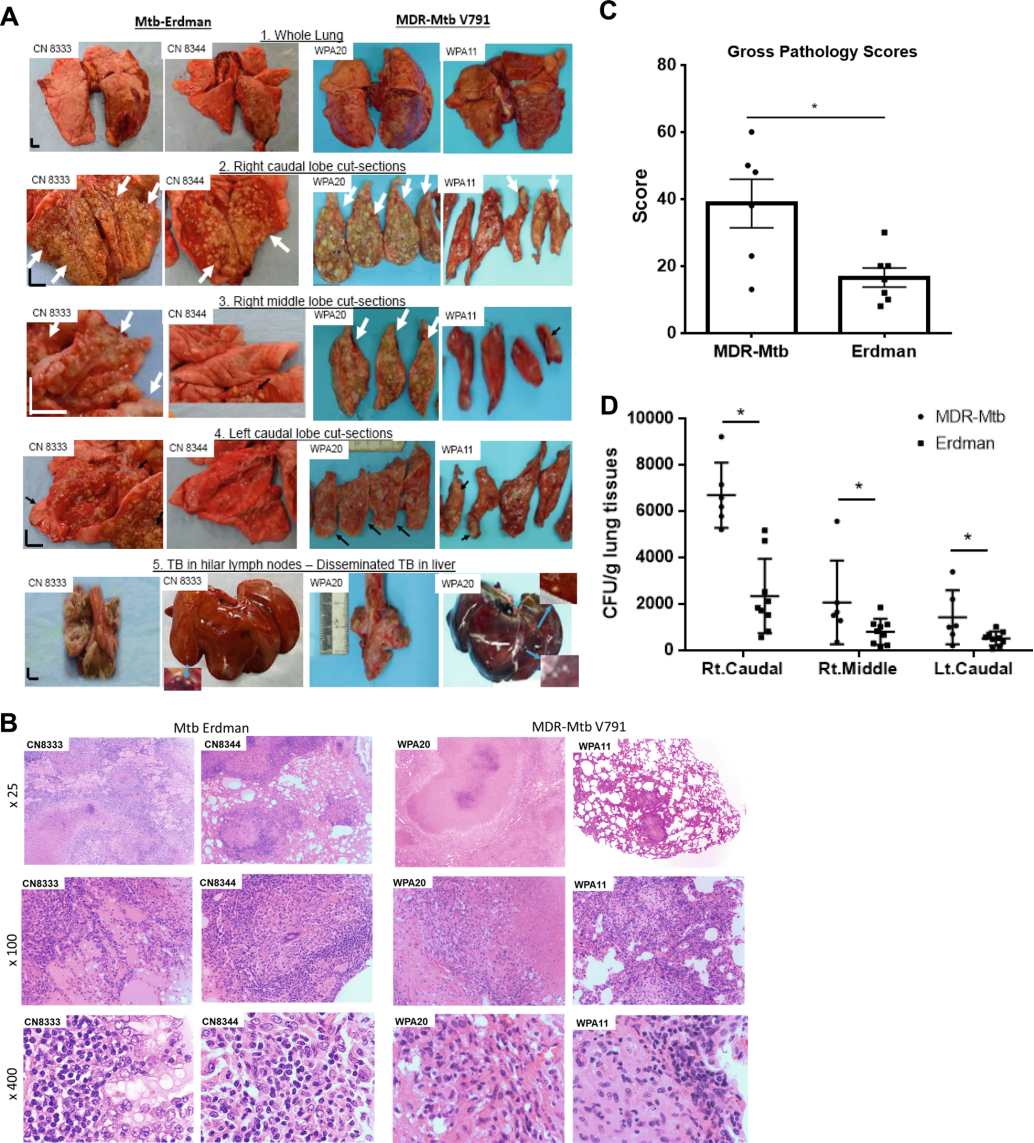
MDR-Mtb induced typical lung TB pathology similar to the Erdman strain after a high-dose 500-CFU infection of nonhuman primates. **a** Representative digital images of the whole lung (panel 1), sections of the right caudal (panel 2), right middle (panel 3), and left caudal (panel 4) lung lobes and the hilar lymph nodes/liver (panel 5) from MDR-Mtb-infected and Erdman-Mtb-infected macaques are shown. Representative MDR-Mtb-infected and Erdman-Mtb-infected macaques with both severe and less severe TB lesions are displayed, with the ID number of the representative monkeys indicated in the upper-left corner. WPA11 served as a less severe example despite moribund related to an overreaction to a transient IL2 treatment. Note that the lung lobes of Erdman-Mtb-infected macaques were opened, whereas those of MDR-Mtb-infected animals were sliced to adhere to the more stringent safety protocols. TB lesions could be adjudged based on the examples indicated by the white arrows demonstrating the presence of caseation pneumonia or extensive coalescing granulomas and by the examples indicated by the small arrows demonstrating less coalescing or noncoalescing granulomas. The vertical/horizontal bars at the bottom left represent the 1-cm scale derived from the fluorescence rulers of each original photo, including the sliced sections. In panel 5, which shows the liver, enlarged views of granulomas from selected areas (blue arrows) are shown in the upper or lower corners. Lungs and other organs were obtained during the necropsies that occurred 2.5–4 months after infection with Mtb. **b** Representative histopathological images of both the severe and less severe TB lesions in representative MDR-Mtb-infected and Erdman-Mtb-infected macaques. H&E stained sections of typical tubercles in the right caudal lobes taken from two representative macaques from each group, with the macaque ID number and magnification indicated in each image. The severe TB lesions from the MDR-Mtb-infected macaque (WPA20) and the Erdman-Mtb-infected macaque (CN8333) are characterized by widespread necrosis and tissue destruction. Many polymorphic leukocytes, epithelioid cells, macrophages, and degenerative or necrotic cells are seen in the edges of the tubercles. There was insufficient infiltration of lymphocytes. The less severe TB lesions shared by the MDR-Mtb-infected macaque (WPA11) and Erdman-Mtb-infected macaque (CN8344) were generally characterized by less necrotic tubercles, which were mainly composed of epithelioid cells, giant cells, fibroblasts, and macrophages. There were notable infiltration zones of lymphocytes, plasma cells, and macrophages around the tubercles. Note the modest amount of inflammatory effusion seen in the alveoli near the tubercles. **c** Mean gross pathology scores ± SEM for MDR-Mtb-infected macaques (*n* = 6) and Erdman-Mtb-infected animals (*n* = 9). The scores were calculated as previously described. Differences between groups were analyzed, but we cannot claim any biological significance due to the existence of different endpoints. In this study, we intended to assess the similarity of MDR TB to DS TB rather than to compare the extent/severity. Most MDR-Mtb-infected macaques received adalimumab near the endpoint. Erdman-Mtb-infected macaques were euthanized ~1–2 months earlier than MDR-Mtb-infected macaques due to budget constraints. **d** High levels of MDR-Mtb burdens in the lungs are associated with the pathology of MDR-TB. The CFU counts per gram equivalent to a milliliter of lung tissue homogenates of the right (Rt) caudal, right middle, and lower left lung lobes collected at the endpoints are shown. To estimate the lung infection levels, the CFUs of MDR-Mtb in lung lobes at the endpoint (at ~4.5 months) were aligned with those of Erdman-Mtb in lung lobes at 2–3 months. We conducted a statistical analysis to estimate the differences, but we cannot claim any biological significance due to the existence of different endpoints and other factors such as adalimumab (Supplementary Figure)

Gross pathological analysis of representative macaques from the MDR-Mtb and Erdman groups showed that MDR-Mtb-infected macaques could exhibit both severe and moderate forms of TB lesions, as observed 2–3 months after infection with a high dose (500 CFU) of the Erdman strain (Fig. 5a). Like Erdman-TB, the severe form of MDR-TB was characterized by caseation pneumonia or extensive coalescing granulomas in the right caudal lung lobe (infection site, Fig. 5a, panel #2); apparent dissemination of TB lesions to the right, middle, and left caudal lung lobes (Fig. 5a, panels #3, #4); and enlarged hilar lymph nodes with caseation (Fig. 5a, panel #5). Notably, there was dissemination of MDR-TB to extrapulmonary organs, including the liver, as observed in severe Erdman-TB (Fig. 5a, panel #5). Concurrently, the less severe form of MDR-TB lesion was mostly confined to the infection site in the lung (Fig. 5a).

Overall, the lesions of MDR-TB histopathologically resembled those of Erdman-TB (Fig. 5b). Severe MDR-TB lesions, similar to severe Erdman-TB lesions, were characterized by widespread tissue destruction and necrosis, with infiltration of many polymorphic leukocytes, epithelioid cells/macrophages, degenerative, and necrotic cells on the edges of necrotic TB granulomas. There was insufficient infiltration of lymphocytes in the infiltrating zone (Fig. 5b). In summary, there were no significant histopathological differences between less severe TB lesions induced by the MDR-TB and Erdman strains (Fig. 5b).

MDR-TB infection of macaques led to severe gross pathology (Fig. 5c) and high levels of MDR-Mtb burdens in the lungs (Fig. 5d) at the endpoints. The pathology and bacilli levels detected at ~4.5 months of MDR-TB infection appeared indistinguishable in nature from those detected at ~2–3 months of Erdman-TB infection (Fig. 5c, d). We conducted statistical analyses to determine the differences, but we cannot claim any biological significance due to differences in the endpoints. Erdman-infected macaques were euthanized earlier due to budget constraints. Our study aimed to obtain information regarding the overall similarity of infections with MDR-Mtb and Erdman-Mtb, rather than comparing the extent and severity, especially given the short-term nature of the study.

Thus, these results suggested that MDR-Mtb V791 infection could induce typical clinical disease and TB pathology similar to that induced by the Erdman strain after high-dose 500 CFU infection of NHPs.

## Discussion

The current study was the first to comparatively investigate pulmonary infection events and innate and adaptive immune responses after infection of macaques with MDR-TB and control DS Erdman strains. Our work demonstrates that MDR strain burdens in the airway are significantly higher than those of the DS Erdman strain soon after pulmonary exposure. These results imply that MDR Mtb V791 can readily undergo airway transmission via the aerosol route. Such early productivity after MDR-Mtb infection upregulated the expression of caspase 3, an apoptotic/cell death surrogate marker, in macrophages/monocytes and induced substantial innate-like IFN-γ and TNF-α responses in the CD3-lymphocyte population and the Mtb phospho antigen-specific γ δ T-cell subset, respectively. Concurrently, broad adaptive responses of T-cell subpopulations producing Th1, Th17, Th22, and CTL cytokines were detected after primary MDR-Mtb infection. Furthermore, MDR-Mtb bacilli, like the Erdman control bacilli, can readily induce typical TB disease that is characterized by weight loss, lymphocytopenia, high levels of lung Mtb burdens, and severe TB pathology.

MDR-Mtb bacilli might replicate productively in the pulmonary compartment upon airway exposure, as the CFU counts in the BALF of MDR-Mtb-infected macaques were significantly higher than those in the BALF of Erdman-infected animals. The results also suggest that MDR-Mtb may possess the capacity to spread or disseminate via the airway after pulmonary infection. It should be mentioned that TB burdens in tissues may not be comparable between the groups due to differences in the endpoints and other factors. Nevertheless, our studies provide in vivo observations suggesting that MDR-Mtb bacilli may resemble wild-type Mtb strains and readily induce productive infections after pulmonary exposure. Notably, the results of in vitro culture studies suggested that due to mutational fitness, MDR-Mtb strains might not necessarily replicate or infect macrophages more successfully than wild-type or DS Mtb strains^7,8^. Our in vivo findings now suggest that MDR-Mtb can maintain replication or infection capabilities in macaques.

The MDR-Mtb infection of macaques appears to elicit more apparent innate-like effector responses of TNF-α-producing CD3 lymphocytes and IFN-γ-producing Vγ2Vδ2 T cells than infection with the control Erdman strain. These robust responses may be driven by the higher CFU burdens in the airway during the primary MDR-Mtb infection than during Erdman-Mtb infection. The upregulated production of TNF-α in the context of increased caspase 3 may favor the ability of infected macrophages/monocytes to undergo apoptosis and the development of innate immunity against the Mtb infection^31,32^. Notably, the effector functions producing TNF-α and IFN-γ may represent appreciable levels of anti-TB immune responses during MDR-Mtb infection, as these two cytokines play important roles in protection against TB^33,34^. TNF-α-producing CD3 lymphocytes likely include protective NK cells or other innate immune cells. It is also noteworthy that Mtb phospho antigen-specific Vγ2Vδ2 T cells exist only in humans and NHPs, and accumulating evidence suggests that this dominant γδ T-cell subset is one of the protective components against Mtb infection^20,26,35^.

Conventional αβ T cells develop broad adaptive responses, producing Th1, Th2, Th17, Th22, and CTL cytokines during MDR-Mtb infection. Such broad T effector responses that produce IFN-γ, IL-17, IL-22, and perforin appear to be transiently more robust in week 3 and are still appreciable at week 8 when compared with those that occur during infection with the control Erdman strain. The initially high levels of adaptive responses are consistent with the upregulated innate-like production of TNF-α and caspase 3. The apoptosis mediated by the caspase 3 TNF-α pathway may in turn facilitate the cross-presentation of Mtb antigens for the efficient initiation of adaptive T-cell immune responses. Given the possibility that Th1^19,36^, Th17^37^, Th22^38^, and CTL^39^ subpopulations protect against Mtb infection^40^, the production of cytokines by T effectors after high-dose primary MDR-Mtb infection may otherwise represent protective responses when they develop after a natural low-dose Mtb infection.

The results of our immune studies suggest that MDR bacilli can act like DS Mtb manipulating the host responses to support the survival and spread of the bacilli. MDR-Mtb infection clearly stimulates broad immune responses, but these vigorous MDR-Mtb-induced responses fail to control the infection. Instead, these potentially overreactive or inflammatory responses may play a role in the development of severe TB lesions that enhance airborne Mtb transmission^10^.

Similar to Erdman-Mtb, MDR-Mtb can efficiently infect cynomolgus macaques and induce typical TB disease and the development of severe TB lesions. This finding is not unexpected because MDR-Mtb burdens in the airway were considerably higher than those of the control Erdman-Mtb. It is noteworthy that our focus here was to assess the similarity between MDR-TB and DS-TB and not to compare the extent or severity, which would have been impossible due to different endpoints between the two groups. As previously described, the high-dose Mtb infection of macaques (500 CFU) inevitably results in severe TB despite the development of robust immune responses^21,29^. Overall, compared with the Erdman-Mtb-infected macaques, the MDR-Mtb-infected macaques had higher BALF CFU counts, which were associated with higher frequencies of IFN-γ+ T cells and more severe TB. In addition, high BALF CFU counts were associated with severe pathology, although we cannot claim any statistical significance due to the longer duration of MDR-TB. The results suggest that selection for the MDR-Mtb mutant does not alter the virulence of the bacilli, despite the possible existence of fitness costs.

Thus, our results in a macaque model suggest that primary MDR-TB appears to be similar to or more pronounced than typical TB in terms of bacterial burdens in the airway, the innate/adaptive immune response, and the disease process. Because NHPs are biologically more similar to humans than other species, our data may provide useful information for predicting the course of primary MDR-TB infection after person-to-person transmission. The findings also support the hypothesis that a vaccine or host-directed adjunctive modality that is effective against DS-Mtb may also have an impact on MDR-Mtb.

## Materials and methods

### Strains, animals, and infection

The *M. tuberculosis* Erdman strain was provided by Dr. Bill Jacobs at the Albert Einstein College of Medicine and Dr. Mike Brennan at the U.S. Food and Drug Administration, and it was used as the DS control. The clinically isolated *M. tuberculosis* V791 strain was provided by the ABSL-III lab of Wuhan University and was proven to be an MDR-Mtb strain because it was resistant to at least RIF, INH, OFX, and SM. Research protocols for experiments involving NHPs were evaluated and approved by the Institutional Animal Care and Use Committees at both the American and Chinese institutions. Likewise, experiments involving Erdman-Mtbor MDR-Mtb were evaluated and approved by the Institutional Biosafety Committees at both the American and Chinese institutions.

Four- to eight-year-old Chinese cynomolgus macaques with 3- to 5-kg body weights were used in this study. Via bronchoscope-guided dissemination in the right caudal lung lobe, nine macaques were infected with 500 CFU Erdman-Mtb^41^, and six macaques were infected with 500 CFU MDR-Mtb. The number of bacilli delivered to each macaque was confirmed by determining the CFU counts of diluted Mtb inoculum on 7H11 BD. Blood and BALF were collected from these macaques after infection for the subsequent experiments according to our previously described protocol^39^. All animals were maintained and used in accordance with the guidelines of the institutional animal care and use committees. This study was specifically reviewed and approved by an ethics committee atour institute. Animals were anesthetized with 10 mg/kg ketamine HCl (Fort Dodge Animal Health, Fort Dodge, IA) i.m. prior to blood sampling. EDTA-anti-coagulated blood was collected to obtain the lymphocytes^19,21^. For BALF sampling, after overnight fasting, animals were tranquilized i.m. with 1–2 mg/kg xylazine (Ben Venue Laboratories, Bedford, OH) and 10 mg/kg ketamine HCl and then given 0.05 mg/kg atropine (Phoenix Scientific, Inc., St. Joseph, MO) i.m. as an anticholinergic; then BALF was collected while the animals were restrained in an upright position.

### Isolation of lymphocytes from blood and BALF

Lymphocytes were isolated according to the protocols described previously^21^. Briefly, peripheral blood mononuclear cells (PBMCs) were isolated from freshly collected EDTA-treated blood by Ficoll-Paque PLUS (Amersham, NJ) density gradient centrifugation. Fresh BALF was filtered through 40-μm cell strainers (BD) and centrifuged for 5 min at 1500 rpm. The cell pellets were treated with 5 ml of RBC blood lysis buffer (Sigma-Aldrich) for 10 min or washed once with 5% fetal bovine serum phosphate-buffered saline (FBS–PBS) after the suspension became clear. Due to limited amounts of cells in BALF at a single time point, we performed the CFU determination and functional analyses of multiple parameters separately.

### Flow cytometry analysis and antibodies

The following antibodies were used for culturing or surface and intracellular cytokine staining (ICS) for flow cytometry: CD28 (CD28.2, BD), CD49d (9F10, BD), CD3-PB (SP34-2, BD), CD4-BV510 (L200, BD), CD8-PB (RPA-T8, BD), IFN-γ-APC (4S.B3, BD), IFN-γ Brilliant Violet 711 (4S.B3, Biolegend), TNF-α-PE (MAb11, BD), TNF-α-PE-Cy7 (MAb11, BD), IL-17-PE (eBio64CAP17, eBioscience), IL-22-biotinylated (anti-human IL-22, RD), Streptavidin-Pacific blue (Invitrogen), Perforin-biotinylated (Pf-344, Mabtech), Caspase 3-AF647 (C92-605,BD), and anti-Vγ2-FITC (7A5, Pierce).

After staining, the cells were fixed and subjected to flow cytometry. Lymphocytes were gated based on forward- and side-scatters, and at least 40,000 gated events were analyzed using Summit Data Acquisition and Analysis Software (DakoCytomation).

### Direct ICS and conventional ICS assays

Direct ICS was performed according to the protocol previously described^19,41^. Briefly, PBMCs or lymphocytes from BALF were used in each reaction to measure the ability of T cells and CD3-negative lymphocytes to constitutively produce IFN-γ, TNF-α, IL-17, IL-22, and perforin without Ag stimulation in vitro. Lymphocytes were incubated for 1 h in a medium with CD28 (1 μg/ml) and CD49d (1 μg/ml) monoclonal antibodies (mAbs) in a final volume of 200 μl at 37 °C in a 5% CO_2_ atmosphere, followed by a 5-h incubation in the presence of brefeldin A (GolgiPlug, BD). After a total of 6 h of incubation, the cells were subjected to surface and intracellular staining.

For the conventional ICS assay, PPD or HMBPP antigens were added to the culture and incubated for 6 h in the presence of anti-CD28 and CD49d mAbs before being cultured for 5 h with GolgiPlug, as described above. The cultured cells were then subjected to surface and intracellular staining.

To ensure specific immune staining during direct or conventional ICS, matched normal serum or isotype IgG served as negative controls for staining cytokines or surface markers.

### Determination of bacterial CFU counts from BALF and lung tissues

Five milliliters of 4% NaOH-decontaminated, filtered BALF from each Mtb-infected animal was first treated with 5 ml of RBC blood lysis buffer (Sigma-Aldrich) for 10 min or maintained until the suspension became clear and then washed once with 5% FBS–PBS and directly plated on 7H11 BD as previously described. To measure the bacilli counts in lung tissues, half of the sections taken from the right caudal, right middle, or the left caudal lobes of each animal were subjected to CFU determination after an extensive gross pathologic evaluation was performed. If there were TB lesions in a lobe, half of the lung tissue, containing approximately 50% of the lesions, was excised. If there were no visible lesions in a given lobe, a random half of the tissue was excised for evaluation. Tissue homogenates were serially diluted in PBS and plated on 7H11 BD as previously described. The 7H11 BD were then incubated in a 37 °C incubator for 3 weeks, and the CFU was counted.

### Gross pathological analyses of the TB lesions and the scoring systems used

Gross pathological analyses of the TB lesions were performed as previously described. Briefly, the animals were euthanized with intravenous barbiturate overdose and immediately necropsied in a biological safety cabinet. Standard gross pathological evaluation procedures were followed by a blinded pathologist and his/her associates, with each step recorded and photographed. Lung lobes; the bronchial, mesenteric, axillary, and inguinal lymph nodes; the tonsils; and other major organs were collected and labeled. Multiple specimens were collected from all tissues with gross lesions, and the remaining major organs were harvested. Gross observations including but not limited to the presence, location, size, number, and distribution of lesions were recorded.

The scoring system was used to calculate gross pathology scores for TB lesions, as described in our previous publications.

### Statistical analysis

The statistical analyses were performed using GraphPad Prism software (GraphPad Software, Inc., La Jolla, CA). The data were analyzed by Student’s *t* test (the parametric method) or by the Mann–Whitney test (the nonparametric method). *p* < 0.05 was considered significant. Only *p* values < 0.05 are shown in the text.

## Supplementary information

Gating strategy for IFN-γ producing Vγ2Vδ2 T cells stimulated by PPD from BALF of Erdman infected animal
