# Supplementary figures and images for: Multidrug-resistant tuberculosis (MDR-TB) strain infection in macaques results in high bacilli burdens in airways, driving broad innate/adaptive immune responses
Source: Emerg Microbes Infect. 2018 Dec 12;7:207. doi: 10.1038/s41426-018-0213-z (PMC6290002; doi:10.1038/s41426-018-0213-z)

## Slide 1
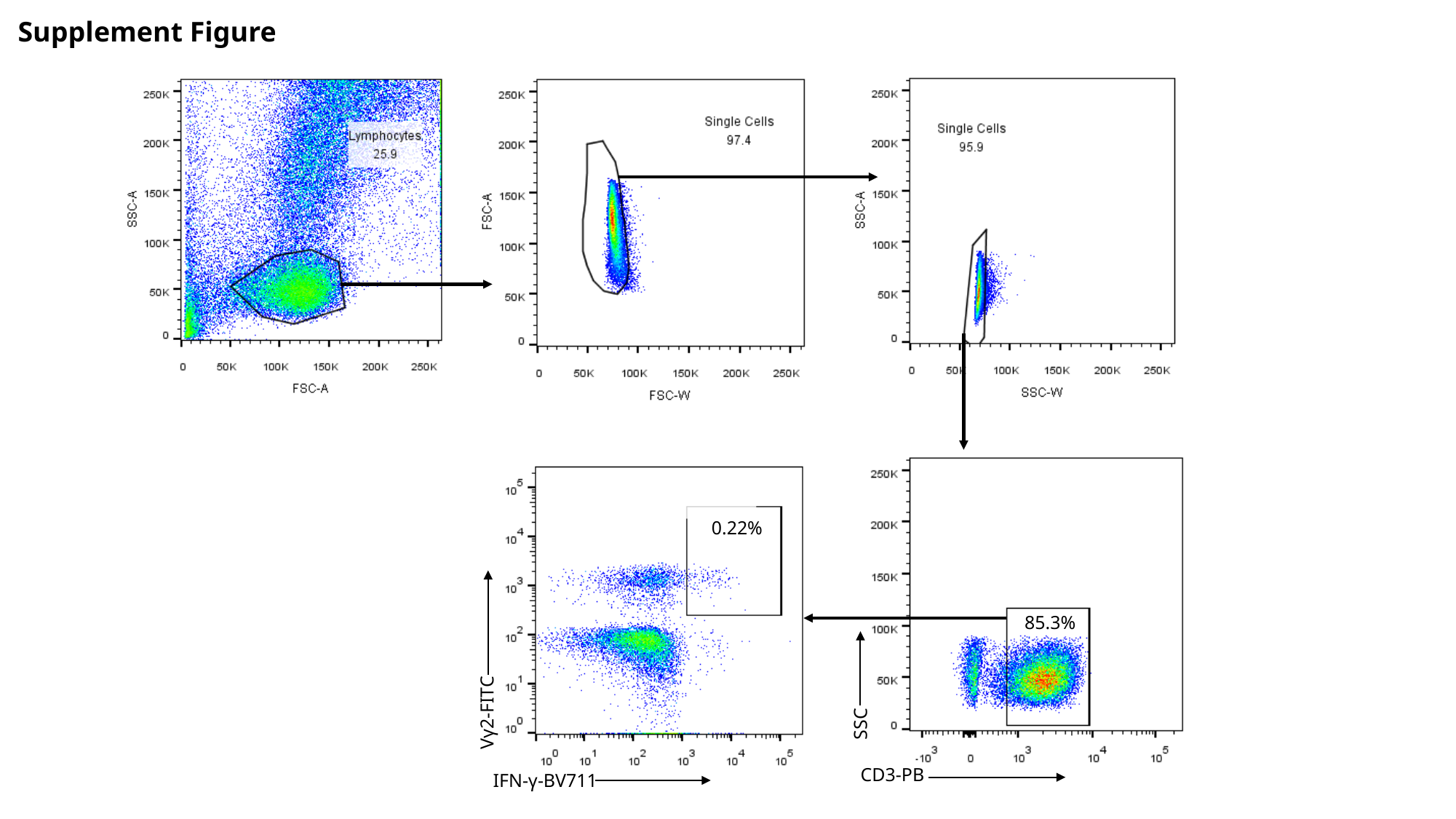

Supplement Figure
0.22%
85.3%
Vγ2-FITC
SSC
CD3-PB
IFN-γ-BV711

Supplement: Supplementary file 1 — Gating strategy for IFN-γ producing Vγ2Vδ2 T cells stimulated by PPD from BALF of Erdman infected animal [file 41426_2018_213_MOESM1_ESM.pptx]
